# Systems Biology Approaches and Applications in Obesity, Diabetes, and Cardiovascular Diseases

**DOI:** 10.1007/s12170-012-0280-y

**Published:** 2012-10-18

**Authors:** Qingying Meng, Ville-Petteri Mäkinen, Helen Luk, Xia Yang

**Affiliations:** Department of Integrative Biology and Physiology, University of California (UCLA), 610 Charles E. Young Dr E., Terasaki Life Sciences Building, Los Angeles, CA 90095 USA

**Keywords:** Metabolic disorders, Obesity, Diabetes, Cardiovascular diseases, Systems biology, Integrative genomics, Functional genomics, Causality inference, Network biology

## Abstract

The metabolically connected triad of obesity, diabetes, and cardiovascular diseases is a major public health threat, and is expected to worsen due to the global shift toward energy-rich and sedentary living. Despite decades of intense research, a large part of the molecular pathogenesis behind complex metabolic diseases remains unknown. Recent advances in genetics, epigenomics, transcriptomics, proteomics and metabolomics enable us to obtain large-scale snapshots of the etiological processes in multiple disease-related cells, tissues and organs. These datasets provide us with an opportunity to go beyond conventional reductionist approaches and to pinpoint the specific perturbations in critical biological processes. In this review, we summarize systems biology methodologies such as functional genomics, causality inference, data-driven biological network construction, and higher-level integrative analyses that can produce novel mechanistic insights, identify disease biomarkers, and uncover potential therapeutic targets from a combination of omics datasets. Importantly, we also demonstrate the power of these approaches by application examples in obesity, diabetes, and cardiovascular diseases.

## Introduction

Common metabolically connected diseases (MetDs) such as cardiovascular disease (CVD), type 2 diabetes (T2D), and obesity impose a substantial health burden worldwide, as demonstrated by the fact that both CVD and T2D are among the top ten leading causes of death in Europe and the United States. As obesity (defined as body mass index (BMI) >30 kg/m^2^) is a key risk factor for both T2D and CVD, the rapidly growing obesity epidemic has further exacerbated the high morbidity and mortality, making an in-depth understanding of the mechanisms of MetDs and the development of novel therapeutic strategies more pressing.

Decades of research on MetDs show that obesity, T2D, and CVD are tightly linked and both genetic and environmental factors contribute to the susceptibility [1]. At the genetic level, hundreds of individual genetic loci are associated with MetDs as shown by recent genome-wide association studies (GWAS) [2•]. It is striking, however, that the genetic loci discovered together only explain a small proportion (typically <20 %) of disease heritability and a large proportion of the loci appear to act through unknown mechanisms [3]. Adding to the genetic complexity, environmental perturbations such as diet, lack of physical activity, and exposure to certain xenobiotic chemicals also increase susceptibility to MetDs but the exact mechanisms remain unclear [4]. Another layer of complexity lies in the intercellular interactions between different cell types, tissues, or organs relevant to MetDs. The above facts call for an improved systems framework to address the missing heritability and molecular mechanisms that underlie MetDs.

The past two decades have witnessed major advances in omics technologies and genome-scale molecular data can now be obtained in diverse experimental and epidemiological settings. A large number of genetic, epigenetic, transcriptomic, proteomic and metabolomic studies of MetDs have already been conducted and numerous datasets at various molecular levels have become available. But collecting information is not enough. A key challenge for the research community is to integrate the sea of data into useful knowledge and insights. To address this critical need, various systems biology approaches have been developed over the past few years to organize the fragmented but valuable information into structures that collectively explain the observed biological phenomena.

**Fig. 1:**
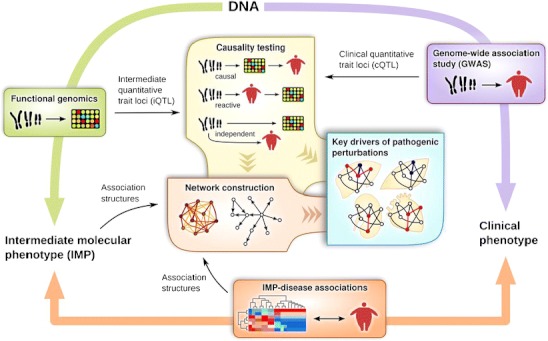
Systems biology strategies that integrate large-scale genetic, intermediate molecular phenotypes (IMPs, primarily gene expression), and disease phenotypes. Traditional genetic association studies such as GWAS identify genetic loci associated with clinical disease phenotypes (cQTLs, *right lavender edge*), which provides causal information but lacks mechanistic insights. Molecular profiling experiments help identify IMPs associated or correlated with disease status (*bottom orange edge*) but the results are purely correlative with no causal information. More recent functional genomics efforts offer mechanistic insights on how DNA variations affect IMPs (primarily gene expression) via the identification of intermediate QTLs (iQTLs; *left lime edge*). By leveraging both iQTL and cQTL and performing statistical testing to differentiate causal, reactive, and independent relationships between IMPs and disease, one can detect putative disease causal genes (*center yellow box*). IMPs, iQTLs, cQTLs, disease phenotypes, and genetic causality can all be fed into various network construction algorithms to reconstruct regulatory networks that inform on mechanisms of IMP and disease regulation (*center orange box*). Higher level integrative approaches that take advantage of multiple methodologies are used to derive key regulatory genes and subnetworks underlying disease development in a tissue-specific fashion (*center blue box*)

Traditional approaches focus on direct correlations between the molecular traits and the clinical diagnosis under investigation, but more advanced integrative or systems biology methodologies can help infer causality, disseminate the regulatory relationships among molecular traits, identify those biological processes or networks that are perturbed, and ultimately pinpoint the key regulatory genes and mechanisms of disease pathophysiology (Fig. 1). In this article, we systematically review the principles, advantages, and limitations of various systems biology approaches available and how these approaches have been applied to MetDs.

### Traditional Approaches-Association and Correlative Analyses

When a particular level of molecular data is generated, the most straightforward approach is to estimate the correlations between the molecular traits and clinical phenotypes. To simplify the discussion, we classify the molecular traits as either DNA, for which variation is typically captured by single nucleotide polymorphisms (SNPs), or intermediate molecular phenotypes (IMPs) that collectively represent the downstream layers such as the transcriptome, epigenome, proteome, or metabolome. Although studies on multiple types of IMPs have been fruitful in the past years [5–8], we mainly focus on gene expression profiling and genetic association studies due to their ubiquitous applications in biomedicine to illustrate the power and limitations of the traditional approaches.

#### Identification of Genes Correlated with MetDs via Gene Expression Profiling

The explosion of gene expression data has made central data repositories such as the NCBI Gene Expression Omnibus (GEO, http://www.ncbi.nlm.nih.gov/geo/) and EMBL-EBI ArrayExpress Archive (http://www.ebi.ac.uk/arrayexpress/) a power house for large-scale systematic meta-analysis, or expression GWAS (eGWAS). It is plausible that disease-related genes exhibit persistent differential expression patterns across multiple studies, and that by scanning through a large data repository related to a given disease condition, these genes can be identified [9, 10, 11•, 12–14]. In a series of studies, Butte and colleagues developed and applied various bioinformatics tools [15–17] to screen GEO datasets for a wide spectrum of human diseases or phenotypes including T2D [11•] and obesity [18]. When conducting an eGWAS across 130 independent experiments that included a total of 1175 T2D case–control microarrays, they identified CD44 as the top differentially expressed gene across studies and experimentally confirmed its role in modulating adipose tissue inflammation, insulin sensitivity, and glycemic control [11•]. Although proven informative and useful, the end products of such analysis are lists of genes correlated with disease status or phenotypes with limited ability to separate non-causal secondary effects from the causal perturbations that trigger and maintain the disease processes.

#### Identification of Genetic Risks of MetDs by Linkage Studies and GWAS

Genetic association studies between genetic markers and disease phenotypes could infer causality to a certain degree under the central dogma that heritable disease risks flow from DNA to other downstream molecular and physiological events. That said, the causality inference is not unambiguous due to potential confounding effects [19]. Before the arrival of comprehensive human genome map and affordable genotyping platforms, linkage studies were extensively used to identify genetic risk loci for MetDs by genotyping sparse genetic markers in human families or F2 crosses of animal models [20–28]. The resulting clinical quantitative trait loci (cQTLs) were then used for fine mapping and positional cloning. However, the linkage blocks represented by the genetic markers were typically large (covering tens or even hundreds of genes), making the identification of candidate genes a labor-intensive and costly process.

In the past few years, human GWAS have achieved great success in uncovering novel genetic risk loci for hundreds of diseases or traits including MetDs and their risk phenotypes [29]. GWAS examine the associations between common genetic variants represented by SNPs and clinical traits or diseases in large human cohorts. As of August 2012, the GWAS Catalog [2•] maintained by the National Human Genome Research Institute (https://www.genome.gov/gwastudies) has collected a total of 6598 SNPs for 677 unique human diseases or phenotypic traits from 1320 different publications. A number of loci have been associated with MetDs−56 for obesity, 48 for T2D, and 38 for CVD. Most of the genetic loci identified were never observed before the GWAS era and many of the candidate genes cannot be explained by current knowledge. For instance, from a recent GWAS of over 80,000 individuals, 13 out of the 23 significant genetic loci identified were novel and 17 appeared to contribute to CAD risk through mechanisms independent of traditional risk factors [3].

Despite the demonstrated power in uncovering novel genetic risk factors, GWAS have limitations. First, as SNP markers on GWAS genotyping panels are largely pre-selected tag SNPs rather than functional SNPs, the discovered loci may not directly represent the functional causal SNPs but may simply be proxies or in linkage disequilibrium with true causal genetic variations. Second, even if the SNP is causal itself, the exact functional consequences of the SNP and the genes affected are not directly identifiable, especially when the SNP is (as 40 % of the significant SNPs are) located in intergenic regions or within introns [30]. The third issue with GWAS is the missing heritability [31]. For example, the largest meta-analysis of GWAS for obesity where ~250 k individuals were surveyed identified a total of 32 significant loci for BMI but together they only contribute to 2-4 % of the genetic variance [32]. Similarly, all 56 established loci identified for T2D together only explain ~10 % of the heritability [33].

The lack of explanatory and predictive power has cast severe criticisms and doubts on GWAS. That being said, GWAS provide first-hand information about the putative causal relationships between genetic variants and clinical diseases traits. In addition, much of the hidden heritability that could not pass the stringent genome-wide significance threshold may lie within the lower tier GWAS signals and can be brought back to light using more advanced integrative methodologies, as discussed later in the review.

### Functional Genomics

Once a genetic locus has been linked to a disease phenotype, the most intuitive step is to search for candidate genes in the neighborhood of the locus. However, tens to hundreds of genes may underlie each cQTL and a large proportion of the GWAS loci lie within intergenic regions, making it difficult to pinpoint the true underlying causal gene and mechanisms. Functional genomics aims to bridge the gap. In principle, genetic loci can impose disease risk via the products of the genome, that is, IMPs such as gene expression, microRNA, DNA methylation, protein levels and downstream metabolites. By linking genetic markers or SNPs to IMPs, functional consequences of genetic perturbations and mechanistic insights can be inferred.

As summarized in our recent review articles [34, 35•, 36], various types of IMPs have been screened in terms of their relationships with genetic variations to produce a set of intermediate molecular QTLs (iQTLs). In particular, the subset of iQTLs that are related to gene expression, denoted by expression QTLs (eQTLs), have been identified in a spectrum of MetD-related tissues or cell types including whole blood, monocyte, lymphocyte, liver, adipose, and muscle in humans [35•] and in a wider coverage of cell/tissue types in animal models or other model organisms [37–39]. Furthermore, large-scale projects such as The Encyclopedia of DNA Elements (ENCODE; http://www.encodeproject.org/ENCODE/index.html) and Genotype-Tissue Expression (GTEx; http://commonfund.nih.gov/GTEx/) are likely to provide a comprehensive characterization of the full functional genome in the future. These iQTL/eQTL studies are able to establish the fundamental genetic regulatory architecture of IMPs, and thus serve as the basis for causality inference between genes and disease phenotypes.

### Causality Inference via Integration of Genetic, Gene Expression, and Disease Phenotypes

Based on the central dogma, heritable information flows from DNA to RNA, to proteins, and then to phenotypic traits. When cQTL and eQTL overlap, that is, a genetic locus or multiple genetic loci are in association with both disease phenotype and the expression levels of a gene, there is a higher probability that the gene associated with the disease-linked genetic loci is also the causal gene. Based on this principle, Meng et al. identified a significant overlap between the cQTL of aortic calcification and the eQTL for gene ABCC6 in a mouse F2 population and experimentally validated ABCC6 as the causal gene for the phenotype [40]. The same principle has been applied to human GWAS studies where disease-associated SNPs are intersected with eSNPs (SNPs associated with gene expression levels under eQTLs) databases to derive candidate genes for MetDs and related phenotypic traits [3, 35•, 41, 42•, 43, 44, 45•, 46•, 47, 48]. The identification of SORT1 as the gene behind the chromosome 1p13.3 locus for LDL cholesterol and CVD is another example of successful causal inference via functional genomics. Altogether three genes – PSRC1, CELSR2, and SORT1 – were located within the adjacency of the 1p13.3 risk locus, but SORT1 was identified as the strongest candidate due to the most significant association between SORT1 expression and the 1p13.3 GWAS SNP in a liver eQTL study [44]. Tested in both transgenic and knockdown mouse models, SORT1 was successfully validated as the causal gene for LDL and CVD via modulation of hepatic lipoprotein export by two groups [49, 50]. In cases where the GWAS locus is within an intergenic region, eSNPs can reflect genetic control of gene expression regardless of the genomic position. For instance, expression levels of *ULK3* are consistently associated with the intergenic CVD locus 15q24 across adipose, monocyte, liver, and blood [35•], thus representing a plausible candidate gene for this locus.

As IMPs can be upstream (i.e., causal), downstream (i.e., reactive), or independent of a disease phenotype, eQTL and cQTL overlap does not directly implicate causality. The three relationships can be formally tested via statistical and mathematical inference by using DNA or genetic variation information as the anchor (Fig. 1). To this end, a likelihood-based causality model selection (LCMS) procedure was developed by Schadt et al. [20]. The LCMS causality test was applied to obesity and atherosclerosis phenotypes in mouse F2 populations and successfully identified hundreds of candidate causal genes for adiposity and aortic lesions [20, 51, 52•]. In the obesity study, perturbation of eight top obesity candidate causal genes for obesity - Zfp90, Lpl, Tgfbr2, C3ar1, Gpx3, Gas7, Lactb, and Gyk - was found to alter adiposity or fat pad mass in knockout or transgenic mouse models via modulation of genes involved in metabolic pathways and a liver network of genes involved in lipid metabolism [51]. In the atherosclerosis study, the top causal genes for aortic lesions were enriched for those involved in inflammatory processes such as lymphocyte activation and B cell receptor signaling [52•]. Knockout mouse model of a candidate causal gene, C3ar1, was found to reduce aortic lesions. In addition, the expression levels of most causal genes in the aortic arch altered accompanying lesion progression in two independent atherosclerosis mouse models. Furthermore, several causal genes overlapped with candidate genes from CVD-related human GWAS. These validation experiments strongly support the validity and power of LCMS in predicting reliable causal genes for complex MetDs.

### Construction of Molecular Networks via Integration of Genetics, IMPs, and Disease Phenotypes

All the methodologies outlined above yield lists of molecular markers that are linked to disease development, but they offer little information on how genes and other IMPs are organized and how they operate together in complex biological systems. Networks have emerged as appealing tools to address this complexity; they depict the active agents in the systems as nodes, and their interactions as edges that connect the nodes. Notably, the edges can represent different types of relationships such as correlation, physical binding, biochemical reactions or transcriptional regulation, thereby transcending the boundaries of conventional statistics.

Networks can be constructed based on curated knowledge (knowledge-driven) or computational modeling of large-scale genomic data (data-driven). Examples of knowledge-driven networks include protein-protein interaction (PPI) networks in the Human Protein Reference Database (http://www.hprd.org/), Biological General Repository for Interaction Datasets (BioGRID, http://thebiogrid.org/), and Ingenuity networks (www.ingenuity.com). These networks can capture literature-supported relationships but are far from being comprehensive and novel relationships or insights will not be covered. On the other hand, data-driven network reconstruction or reverse-engineering approaches systematically and objectively scan through and integrate all data points to uncover novel relationships among IMPs within a cell or a tissue, or even across tissues [53]. Information obtained from correlations, cQTLs, eQTLs, and causality inference discussed above can all be efficiently incorporated and utilized in various network reconstruction approaches (Fig. 1).

Examples of data-driven networks include weighted gene co-expression network analysis (WGCNA) [54, 55], Bayesian network (BN) [56–60], graphical Gaussian models (GGMs) [61–63], and algorithm for the reconstruction of accurate cellular networks (ARACNE) [64]. Although these different methodologies can be applied to various types of common complex diseases, studies on MetDs have primarily employed WGCNA and BN to identify disease-associated key regulatory genes and gene sub-networks [65–69, 70•, 71, 72•].

#### Weighted Gene Co-expression Network Analysis (WGCNA)

WGCNA aims to identify the correlation patterns among IMPs (primarily gene expression traits) across samples involved in a study. The construction of co-expression network starts with a Pearson correlation matrix between all gene pairs, followed by transformation of the correlation matrix into an adjacency matrix [54, 55]. The adjacency matrix is further transformed into a topological overlap matrix based on the direct interactions between genes as well as the indirect interactions with all the other genes [73]. An average linkage hierarchical clustering algorithm is then applied to the topological overlap matrix , which is followed by a dynamic cut-tree algorithm to identify gene modules [74]. Correlations between the principal components of each module and phenotypic traits measured in the same individuals can be calculated to derive informative modules that link to the disease of interest. Alternatively, co-expression networks can be constructed separately for disease cases and controls, and network modules that demonstrate differential network topology and connectivity between cases and controls can be identified [75].

In contrast to simple clustering algorithms where genes are grouped based on the strength of pair-wise correlations, WGCNA searches for higher-level co-regulation structures. Importantly, the gene memberships of a module are determined not only by their direct correlations but also by the similarity in their relationships with the other genes [54, 73]. The network structure derived is hence comprised of more cohesive and biologically more meaningful modules that contain genes with shared regulatory mechanisms, involved in similar biological functions or pathways, or enriched for disease associated genes [44, 54, 65–69, 76–78].

Numerous studies have applied WGCNA to study the molecular mechanisms underlying MetDs or related phenotypes [67, 68, 71, 72•, 76, 79–86]. As exemplified in two parallel studies, Chen et al. and Emilsson et al. identified a co-expression module that is conserved between liver and adipose tissues, conserved between human and mouse, highly enriched for macrophage- and spleen-related inflammatory genes, and linked to various metabolic phenotypes including adiposity, atherosclerosis, and plasma lipids, insulin and glucose levels [65, 66]. This module is termed macrophage-enriched metabolic network (MEMN). Several novel genes in MEMN, including Lactb, Lpl, and Ppm1l, were experimentally confirmed to affect adiposity in knockout and transgenic mouse models [65]. Ghazalpour et al. and Lum et al. identified gene modules related to obesity and diabetes by using gene expression data from liver and whole brain tissues from a mouse cross segregating on metabolic phenotypes. Genes in the obesity-linked liver module are involved in adipogenesis and fatty acid metabolism whereas genes in the obesity- and T2D-associated brain subnetworks are involved in diverse processes including RNA splicing, circadian rhythm, and lipid metabolism. By constructing co-expression networks in six metabolically related tissues in a mouse population with varying T2D susceptibility, Keller et al. identified a cell cycle regulatory module in islets that predicts islet replication and diabetes development [84]. In two studies on a Finnish cohort, Inouye et al. constructed co-expression networks using blood transcriptomic data and identified a lipid-leukocyte module that was highly enriched for inflammatory genes and significantly linked to over 80 serum metabolites including lipoprotein subclasses, lipids, and amino acids, thereby playing an important role in connecting inflammation, metabolism, adiposity, and atherogenesis [71, 72•]. All these examples substantiate the power of WGCNA in identifying novel genes and mechanisms that contribute to MetDs.

#### Bayesian Network (BN)

Although WGCNA is highly informative for deriving the overall organization of genes or other IMPs and for linking particular co-expression modules to disease phenotypes, the detailed relationships among genes within a module or between modules can be less descriptive. Graphical network modules such as BNs can provide more granular views of the interactions and directionalities between genes. BNs define a partitioned joint conditional probability distribution over all nodes (genes or other IMPs) in a network where the probability distribution of states of a node depends only on the states of its parent nodes [87]. Therefore, BNs are probability-based directed acyclic graphs. The conditional probabilities reflect not only relationships between genes, but also the stochastic nature of these relationships. Due to computational constraints, thousands of plausible BNs can be generated using Monte Carlo Markov chain (MCMC) simulations [88] rather than an exhaustive search for all possible network structures. The posterior probability of each BN model given observed data can be calculated using the Bayes formula. A consensus BN that contain nodes and edges appearing in a large proportion of all plausible network models is then derived. As probability distributions are bi-directional and can lead to mathematically equivalent structures, it is not possible to infer causal directions between nodes. Fortunately, BN framework can incorporate a variety of prior information, ranging from literature, genetic, transcription factor binding, metabolomics, to proteomic data, to break the symmetry among nodes and infer causal directions [56, 57]. As the BN algorithm imposes heavy computing burden and only conserved nodes and edges across plausible networks are kept, BNs are sparser than co-expression networks and not all genes profiled are included in the BN model.

A number of studies in a variety of species have demonstrated that BNs can capture fundamental properties of molecular interactions in complex systems and can infer mechanisms [44, 56, 57, 78, 89, 90]. In searching for the mechanisms underlying the previously discussed lipid and CVD locus 1p13.3, Schadt et al. found that the three candidate genes adjacent to the locus−SORT1, CELSR2, and PSRC1− are highly connected in liver BNs. In addition, the neighborhood subnetworks of the three genes, particularly that of SORT1, are enriched for genes involved in multiple biological processes relevant to lipid regulation and CVD development, thus providing mechanistic support on the involvement of the candidate genes in CVD [44]. To illustrate how candidate causal genes identified via the LCMS causality test described above interact and affect obesity, causal genes were mapped to a liver BN and they were found to be highly connected in a subnetwork, with the top causal gene Zfp90 being upstream of the other causal genes [20]. In a follow-up validation study, by mapping the liver genes perturbed by the overexpression or knockout of top obesity candidate causal genes to liver BNs, a liver core subnetwork that is highly enriched for genes involved in lipid metabolism and fat cell differentiation pathways was identified, further elucidating the mechanisms underlying obesity development [51].

### High Level Integrative Approaches

As shown in Table 1, each methodology described above provides different levels of information with gradual increase in the power to inform on causal genes, biological processes and mechanisms involved in disease pathogenesis. However, each methodology also carries intrinsic limitations. To maximize our ability to discover novel insights, higher level integrative approaches that take advantage of different combinations of the above-mentioned methods have been recently explored and we highlight two such methodologies.

**Table 1:** Comparison of integrative methodologies discussed in the manuscript

Methodology	Brief description	Information derived	Advantages	Limitations
IMP-disease association or correlation analysis	Association or correlation analysis between IMPs and disease phenotypes	List of differential IMPs between cases and controls or IMPs correlated with quantitative phenotypes	Informative on IMPs co-segregating with disease	No causality information
Linkage studies or GWAS	Association between genetic markers or dense SNPs with disease phenotypes	List of genetic loci associated with disease (cQTLs)	Implicates potential causal role of genetic loci	Confers little information on underlying genes and mechanisms
Functional genomics	Association between genetic markers or dense SNPs with IMPs	IMPs that are associated with genetic loci	Infers functional consequences of genetic loci on IMPs; inform on molecular mechanisms	No information on disease relevance
Causality test	Testing causal, reactive, and independent relationships between IMPs and disease by anchoring at shared genetic loci (cQTL/eQTL overlap)	List of genes tested causal for the disease	Inform on candidate causal genes for disease	Statistical inference only and validation needed; little mechanisms
WGCNA network modeling	Organizing IMPs into co-regulated network modules based on correlations between IMPs	Global overview of co-regulation or co-expression structure of IMPs and modules associated with disease phenotypes	Inform on disease mechanisms	Mainly a co-regulation structure but with little regulatory mechanisms
BN modeling	Integrating multiple levels of IMPs to define regulatory relationships	Graphical model depicting detailed interactions and relationships between IMPs	Inform on regulatory mechanisms between IMPs	Computationally intensive, sparse, no feedback loops
Network-driven higher level integration	Integrate network models with GWAS, functional genomics, causality, and IMP profiling to identify key driver genes and subnetworks associated with disease	Key driver genes and subnetworks associated with disease	Prioritize genes and provide mechanisms	Although most informative given higher amount of data incorporated, still hypothesis generating in nature and warrants experimental validation

#### Integration of Disease-Related Gene Sets and Networks

To harness the strengths of data-driven regulatory networks, the information from gene expression profiling, causality testing and GWAS can be overlaid onto co-expression networks and BNs to infer disease mechanisms and key regulatory genes. In a recent study, we identified a consistent differential gene signature comprised of ~1500 inflammation-related genes, termed inflammatome, from 12 tissue-specific gene expression profiling data of 11 different mouse and rat disease models of obesity, diabetes, atherosclerosis, respiratory diseases, autoimmune diseases, inflammation, aging, and sarcopenia [70•]. This inflammatome gene set was integrated with the GWAS catalog and the metabolic disease-related MEMN to confirm the causal nature of the gene signature. By intersecting the inflammatome signature with co-expression networks and BNs constructed from multiple independent datasets, we not only extracted a consistent subnetwork representing the inflammatome signature and detailing gene-gene relationships, but also identified top key regulatory genes based on the BN network topology [70•, 78, 91]. The identification of the common inflammatome signature, its network architecture, and key drivers via this highly integrative approach sheds light on the shared etiology and potential therapeutic targets between MetDs and other common diseases or pathophysiological conditions, a level of mechanistic insights far beyond that of what IMP profiling could offer by itself. Other types of networks such as PPI networks can certainly also be integrated, as exemplified by Mori et al. in a study comparing two mouse strains with different susceptibility to diabetes [92•]. By leveraging tissue-specific gene expression with PPI networks, they identified an inflammation- and immune system-related adipose subnetwork that contributes to the differences in diabetes risk.

#### Integration of GWAS, Functional Genomics, Biological Pathways, and Data-Driven Networks

In order to explore the candidate causal genes and mechanisms behind GWAS, several novel functional genomics and network-driven methodologies have been recently developed. By integrating GWAS with functional genomics (primarily eQTLs or eSNPs), Zhong et al. found that eSNPs from T2D-related tissues such as liver and adipose tissues are more enriched for T2D risk SNPs [45•]. Furthermore, by coupling T2D GWAS and eSNPs with biological pathways, candidate causal genes, co-expression networks, and BNs, multiple subnetworks and biological processes such as lipid and fatty acid metabolism, calcium signaling, PPAR signaling, TGF-beta signaling, tight junction, complement and coagulation, antigen processing and presentation, and fat cell differentiation were found to be enriched for T2D genetic risks [45•, 46•, 93]. It is of note that most genes in the significant pathways or subnetworks only showed modest association in GWAS and therefore were missed by the traditional GWAS analysis. These results support the hypothesis that a large number of genes in relevant biological processes with modest effect sizes, rather than only a handful of individual genes with strong effects, collectively contribute to disease development. In another method, Kang et al. screened hundreds of co-expression networks for genes that were consistently co-expressed with known top T2D GWAS candidate genes as a means to identify novel T2D genes [94•]. These new methodologies not only provide mechanistic explanations for GWAS findings but also demystify a significant amount of the missing heritability.

## Conclusion

Systems biology approaches that leverage genetic, tissue-specific IMP profiling data, and disease phenotypes have evolved rapidly in the past decade. Through their applications in various MetDs in both animal models and human populations, these highly integrative systems biology approaches have unveiled unprecedented insights into disease etiology and uncovered a large number of candidate novel genes, pathways, and subnetworks associated with MetDs. By far, inflammation and immune response related genes and processes have been the most consistent signal across tissue types, across studies, and across MetDs, and thus convincingly represent a key shared component of MetDs. The systems integration of tissue-specific molecular data also revealed many tissue- and disease-specific processes, such as liver-centric lipid metabolism and transport pathways for obesity and CVD; liver- and adipose-specific oxidative phosphorylation, fatty acid oxidation, PPAR signaling, fat cell differentiation for obesity, insulin resistance, and T2D; liver-specific glucagon signaling and islet-specific cell cycle regulation for T2D; and circadian rhythm and RNA splicing processes in brain for obesity and T2D.

These findings highlight how tissue-specific gene networks and their cross-tissue interactions, rather than individual genes, mediate MetD etiology. It is therefore critical to shift from a traditional view of disease mechanisms as independent actions of individual genes to a network view, where a large number of genes coordinately define a particular network state in individual tissues and the interactions of gene networks in multiple tissues ultimately lead to MetD onset.

### Future Directions

Although proven predictive and informative, the existing systems biology methodologies are far from being comprehensive and accurate. Further refinement of existing methods and development of more advanced approaches are thus warranted. First of all, incorporation of next generation sequencing, DNA methylome, microRNA, metabolomics, and other types of data into the systems biology framework has become more pressing than ever, as such data are being rapidly generated and poured into data depositories in the past couple of years. Although some of the existing methodologies can be easily adapted for additional data types, innovative approaches guided by biological insights are still in great need. For instance, based on the regulatory relationships across data types, it is necessary to develop methodologies that leverage multiple levels of IMPs simultaneously to construct more sophisticated networks such as co-regulatory microRNA-gene-metabolite networks.

Second, the involvement of multiple cell types, tissues, and organs in MetDs demands methodologies that explore cross-tissue interactions. As demonstrated elegantly in a recent study by Dutta et al., communications and signaling transductions across multiple critical tissues including heart muscle, sympathetic nervous system, bone marrow niches, and spleen are all involved in the enhanced inflammatory response after MI to induce atherosclerosis acceleration [95•]. This type of relationship can only be revealed when data integration reaches organism-wise systems level. Although the construction of cross-tissue networks has been sporadically attempted [84, 96], such efforts have to be further expanded to increase tissue coverage and to develop more efficient methodologies.

Third, most of the current methodologies capture static information that only represents snapshots of disease status at a given time. Dynamic models that take IMP data generated from time-course experiments are therefore needed to capture the dynamic nature of disease progression. All these different levels of technical and biological challenges have to be properly addressed in the future to allow a full dissemination of MetD etiology. Only when a comprehensive understanding is achieved, can effective diagnostic, preventative, and therapeutic strategies toward these disabling and deadly diseases become a reality.
